# Supplementary material for: An engineered thermal-shift screen reveals specific lipid preferences of eukaryotic and prokaryotic membrane proteins
Source: Nat Commun. 2018 Oct 12;9:4253. doi: 10.1038/s41467-018-06702-3 (PMC6185904; doi:10.1038/s41467-018-06702-3)
Supplement: Supplementary file 1 — Supplementary Information [file 41467_2018_6702_MOESM1_ESM.pdf]

Name	SLC gene family	T_m (°C) Detergent-solubilized membranes	T_m (°C) Purified fusion	T_m (°C) Purified fusion + lipid (*)
Bird FLVCR2	SLC49	32.4 ± 0.4	22.9 ± 0.5	40.2 ± 0.8 (CHS)
Human NHA2	SLC9	38.8 ± 2.0	30.4 ± 0.4	50.5 ± 0.3 (Brain lipids)
Mouse NHA2	SLC9	40.7 ± 0.6	32.0 ± 0.8	44.0 ± 0.6 (Brain lipids)
Plant CMP Sia	SLC35	31.8 ± 0.3	23.2 ± 0.3	30.3 ± 0.7 (CHS)
Human CMP Sia	SLC35	37.1 ± 0.3	25.2 ± 0.6	47.5 ± 0.5 (CHS)
Rat GLUT5	SLC2	33.5 ± 0.3	23.0 ± 1.0	35.6 ± 0.6 (Brain lipids)
Human GLUT 1	SLC2	39.1 ± 0.3	27.2 ± 1.3	58.0 ± 0.7 (Brain lipids)
AmtB		77.9 ± 0.3	72.6 ± 4.2	
$ASBT_{NM}$		52.6 ± 0.4	39.8 ± 0.5	
EmrD		34.8 ± 0.7	27.9 ± 1.3	
GlpG		63.9 ± 0.5	51.3 ± 0.5	
GlpT		35.9 ± 0.7	33.5 ± 1.2	
Mhp1		41.0 ± 0.3	44.8 ± 0.4	
LacY		35.7 ± 0.3	36.7 ± 1.5	
NapA		72.1 ± 0.9	71.6 ± 0.5	
NhaA		42.6 ± 1.2	42.4 ± 0.6	
XylE		41.9 ± 0.4	40.3 ± 0.5	

Supplementary Table 1. Apparent T_m melting temperatures of prokaryotic and eukaryotic membrane proteins before and after purification as determined by GFP-TS using a range of 9 different temperatures that were fitted to a sigmoidal dose-response equation as described in Methods. Each apparent T_m (mean ± s.e.m. of the fit) is the average from two independent experiments. Apparent T_m of purified eukaryotic membrane proteins in the presence of the most stabilizing lipid is also shown; melting curves are shown in **Supplementary Fig. 5, 6, 7, 9 and 10**.

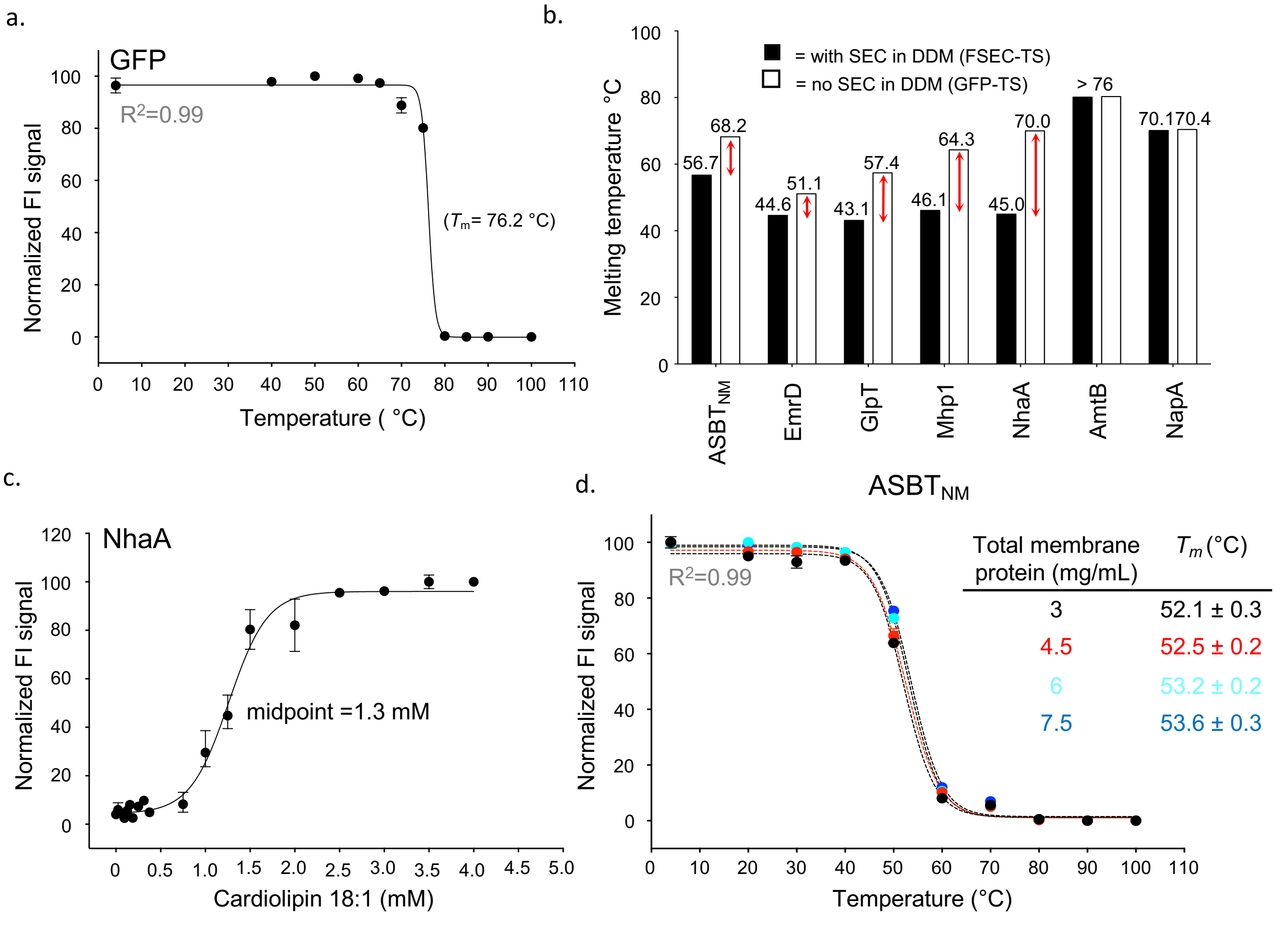

Supplementary Fig. 1. Developing GFP-TS for high-throughput ligand screening a. FSEC-TS melting curve for purified GFP; bars show the range of two technical replicates and the value reported are the mean ± s.e.m. of the fit. **b**. T_m values obtained from the supernatant of DDM-solubilized membrane fusions remaining in solution after heating and centrifugation without β-OG addition (white bars) and those obtained when the same samples were later subjected to hFSEC (black bars). **c**. CL dependent stabilization of NhaA as assessed by GFP-TS.; n = 3 independent titrations and the error-bars show then mean ± s.d of the fit **d**. Apparent T_m values for 1% (w/v) DDM-solubilized ASBT_{NM} containing membranes measured at different final total protein concentrations; bars show the range of two technical replicates, and the values reported are the mean ± s.e.m. of the fit).

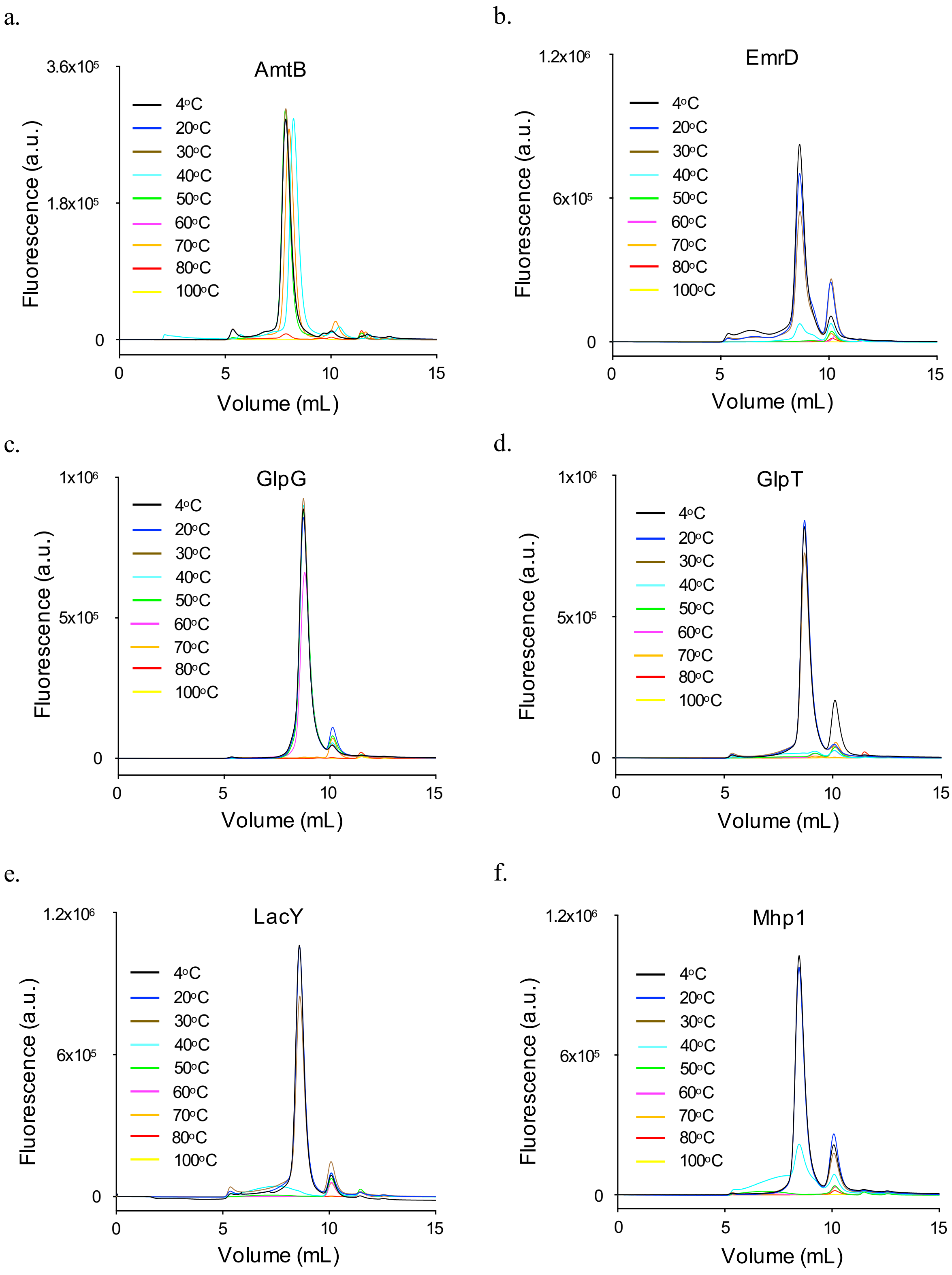

Supplementary Fig. 2. Developing GFP-TS for high-throughput ligand screening. FSEC-TS traces of DDM solubilized bacterial membrane protein GFP-fusions heated at different temperatures after the addition of β-OG as described in Methods: **a**. AmtB, **b**., EmrD, **c**., GlpG., **d**., GlpT **e**., LacY, **f**., Mhp1.

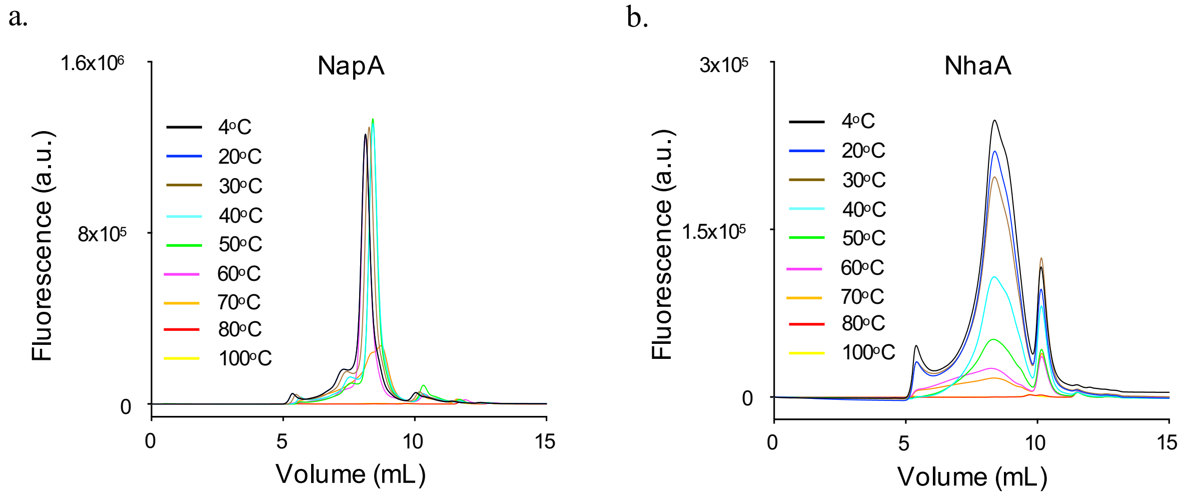

Supplementary Fig. 3. Developing GFP-TS for high-throughput ligand screening. FSEC-TS traces of DDM solubilized bacterial membrane protein GFP-fusions heated at different temperatures after the addition of β-OG as described in Methods: **a.**, NapA and **b.**, NhaA

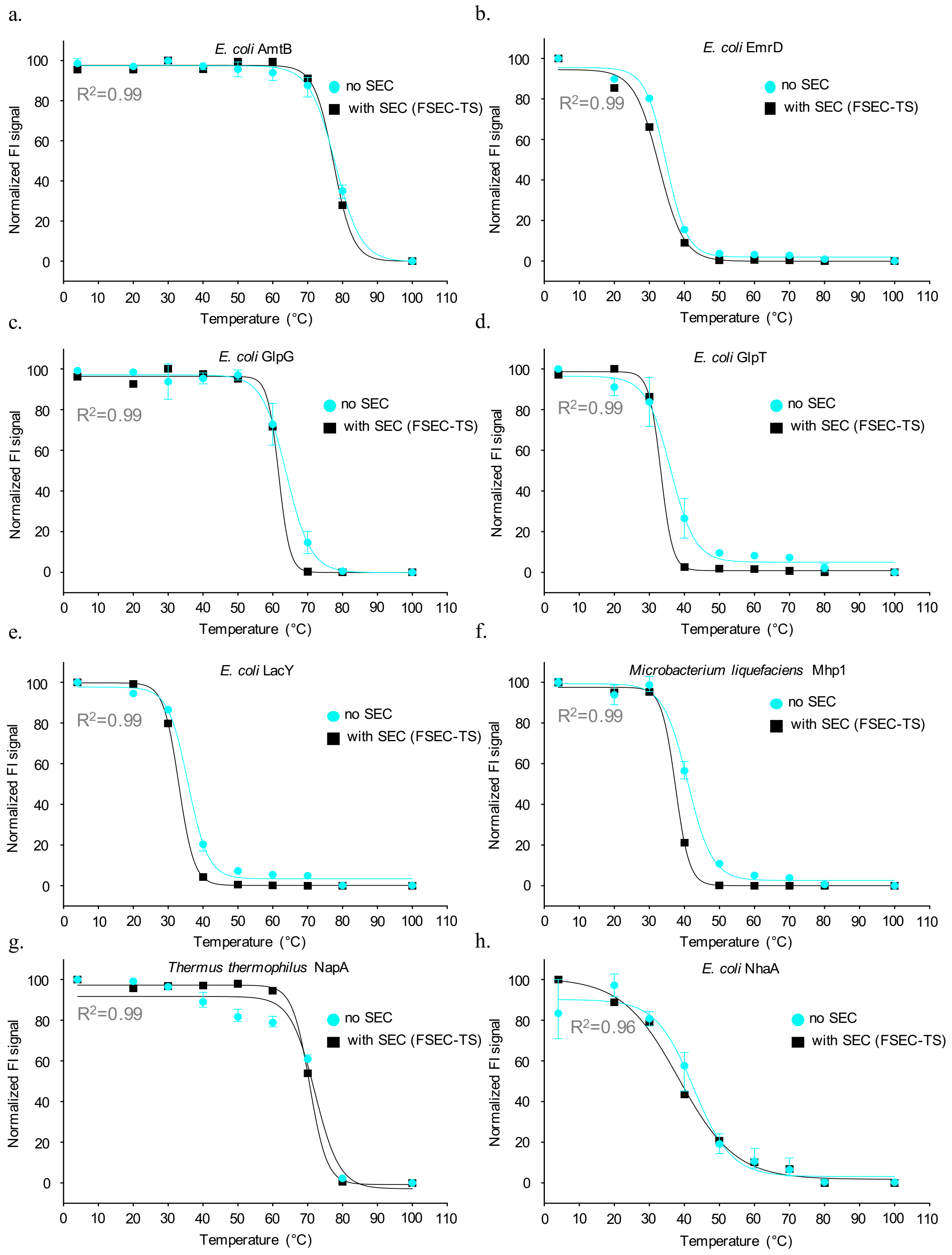

Supplementary Fig. 4 Developing GFP-TS for high-throughput ligand screening. Melting curves from FSEC-TS traces of bacterial membrane protein GFP-fusions shown in **Supplementary Fig. 1** (black curve) and from the fluorescence supernatant, measured on a 96-well microplate spectrofluorometer, of the DDM-solubilized fusions prior to their injection on an HPLC for FSEC analysis (cyan circles). Apparent T_m were calculated using a range of 9 different temperatures that were fitted to a sigmoidal dose-response equation as described in

Methods **a**., AmtB **b**., EmrD **c**., GlpG **d**., GlpT **e**., LacY **f**., Mhp1 **g**., NapA **h**., NhaA (error bars show the range of two technical replicates and the values reported for the apparent T_m are the mean ± s.e.m. of the fit, which were further combined with a separate titration and are tabulated in **Table 1**).

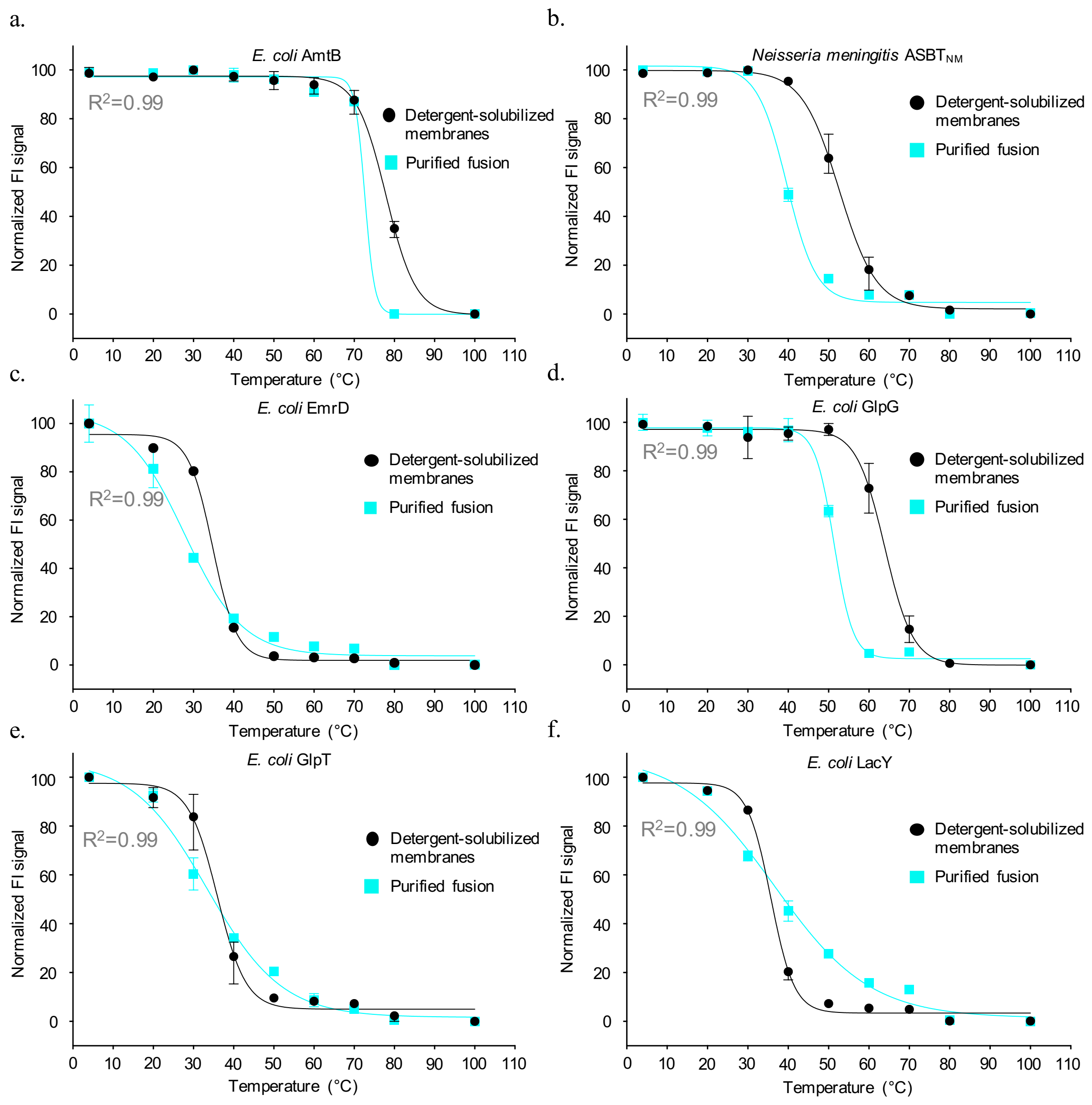

Supplementary Fig. 5 Comparing the lipid stabilization of bacterial vs. eukaryotic membrane proteins. Representative GFP-TS melting curves of crude-detergent solubilized membrane protein fusions (black) and those obtained after purification in DDM (cyan) as described in Methods: **a**., AmtB **b**., $ASBT_{NM}$ **c**., EmrD **d**., GlpG **e**., GlpT **f**., LacY; error bars show the range of two technical replicates and the values reported for the apparent T_m are the mean ± s.e.m. of the fit, which were further combined with a separate titration and are tabulated in **Supplementary Table 1**.

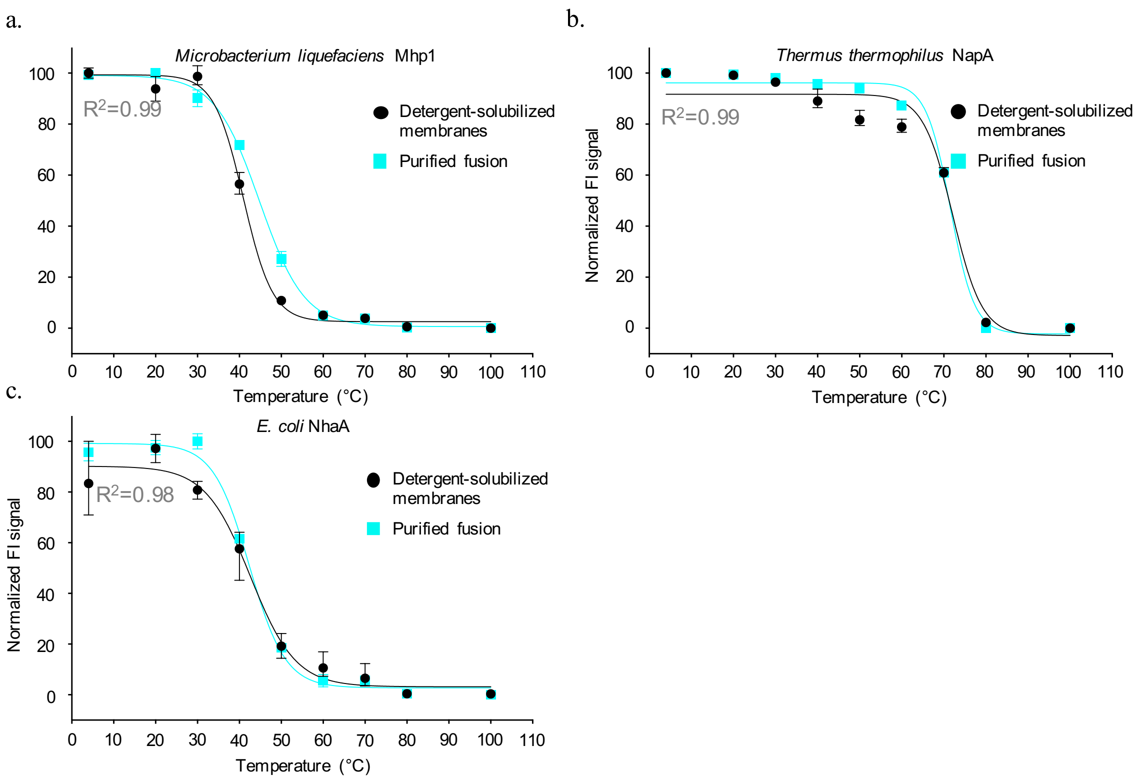

Supplementary Fig. 6 Comparing the lipid stabilization of bacterial vs. eukaryotic membrane proteins. Representative GFP-TS melting curves of crude-detergent solubilized membrane protein fusions (black) and those obtained after purification in DDM (cyan) as described in Methods: **a.**, Mhp1 **b.**, NapA **c.**, NhaA; error bars show the range of two technical replicates and the values reported for the apparent T_m are the mean ± s.e.m. of the fit, which were further combined with a separate titration and are tabulated in **Supplementary Table 1**.

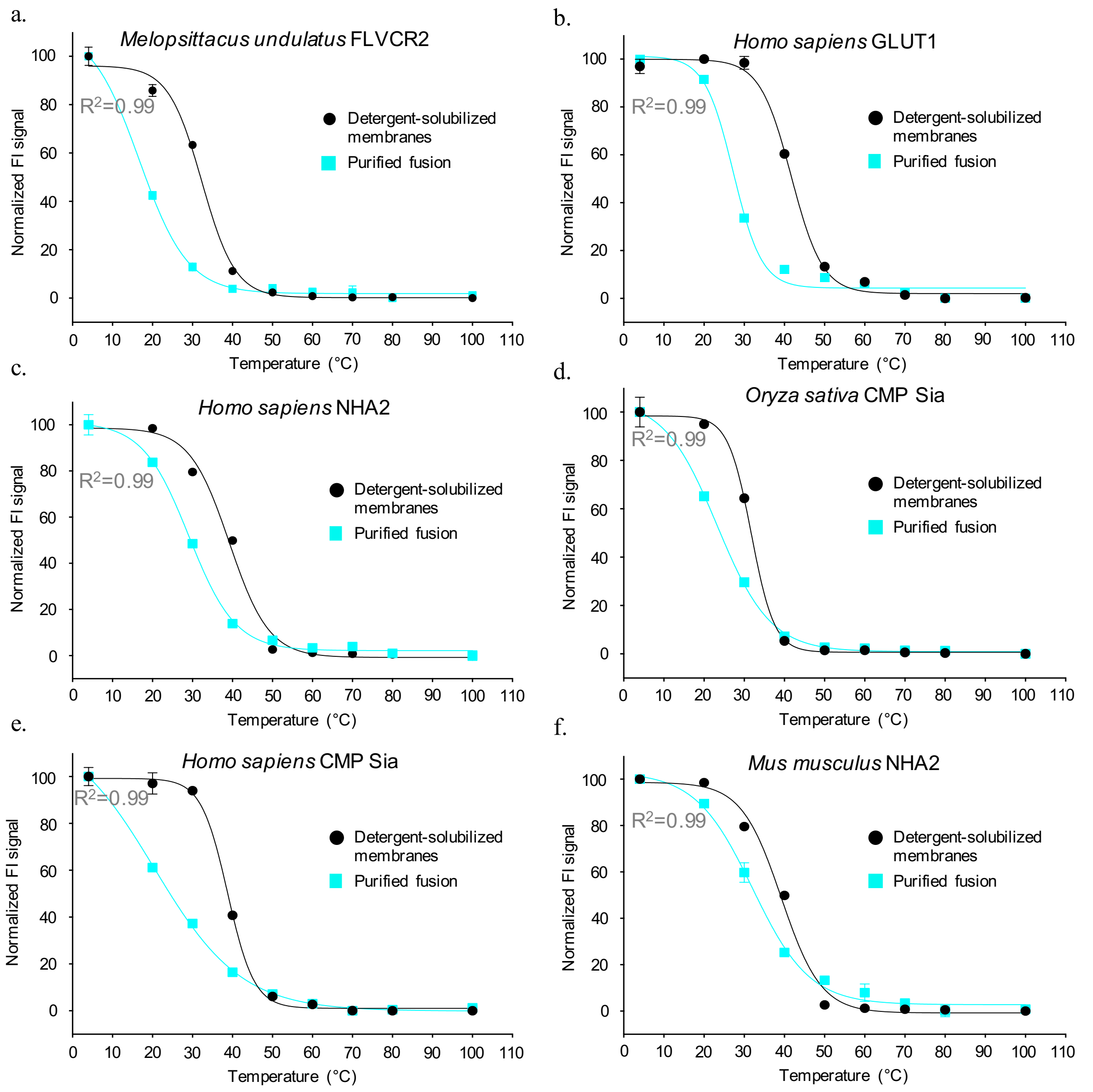

Supplementary Fig. 7 Comparing the lipid stabilization of bacterial vs. eukaryotic membrane proteins. Representative GFP-TS melting curves of crude-detergent solubilized membrane protein fusions (black) and those obtained after purification in DDM (cyan) as described in Methods: **a.**, bird FLVRC2 **b.**, human GLUT1 **c.**, human NHA2 **d.**, rice CMP-Sia **e.**, human CMP-Sia, and **f.**, mouse NHA2; error bars show the range of two technical replicates and the values reported for the apparent T_m are the mean ± s.e.m. of the fit, which were further combined with a separate titration and are tabulated in **Supplementary Table 1**.

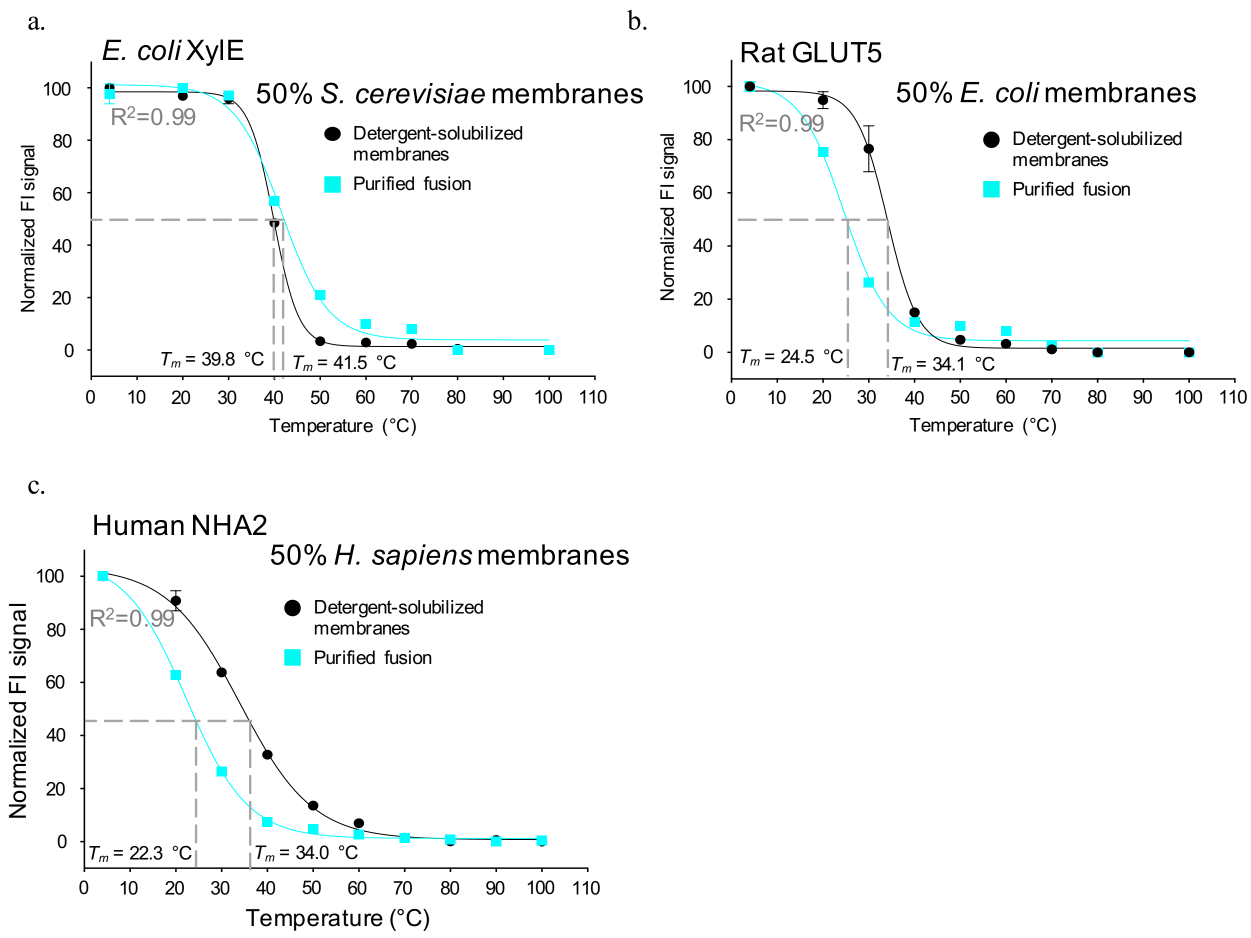

Supplementary Fig. 8 Comparing the lipid stabilization of bacterial vs. eukaryotic membrane proteins. Representative GFP-TS melting curves of crude-detergent solubilized membrane protein fusions (black) and those obtained after purification in DDM (cyan) as described in Methods **a.**, XylE after equal amounts of *S. cerevisiae* detergent solubilized membranes. **b.**, rat GLUT5 after equal amounts of *E. coli* detergent solubilized membranes and **c.**, human NHA2 after equal amounts of *H. sapiens* detergent solubilized membranes; error bars show the range of two technical replicates and the values reported for the apparent T_m are the mean ± s.e.m. of the fit and are tabulated in **Supplementary Table 1.**

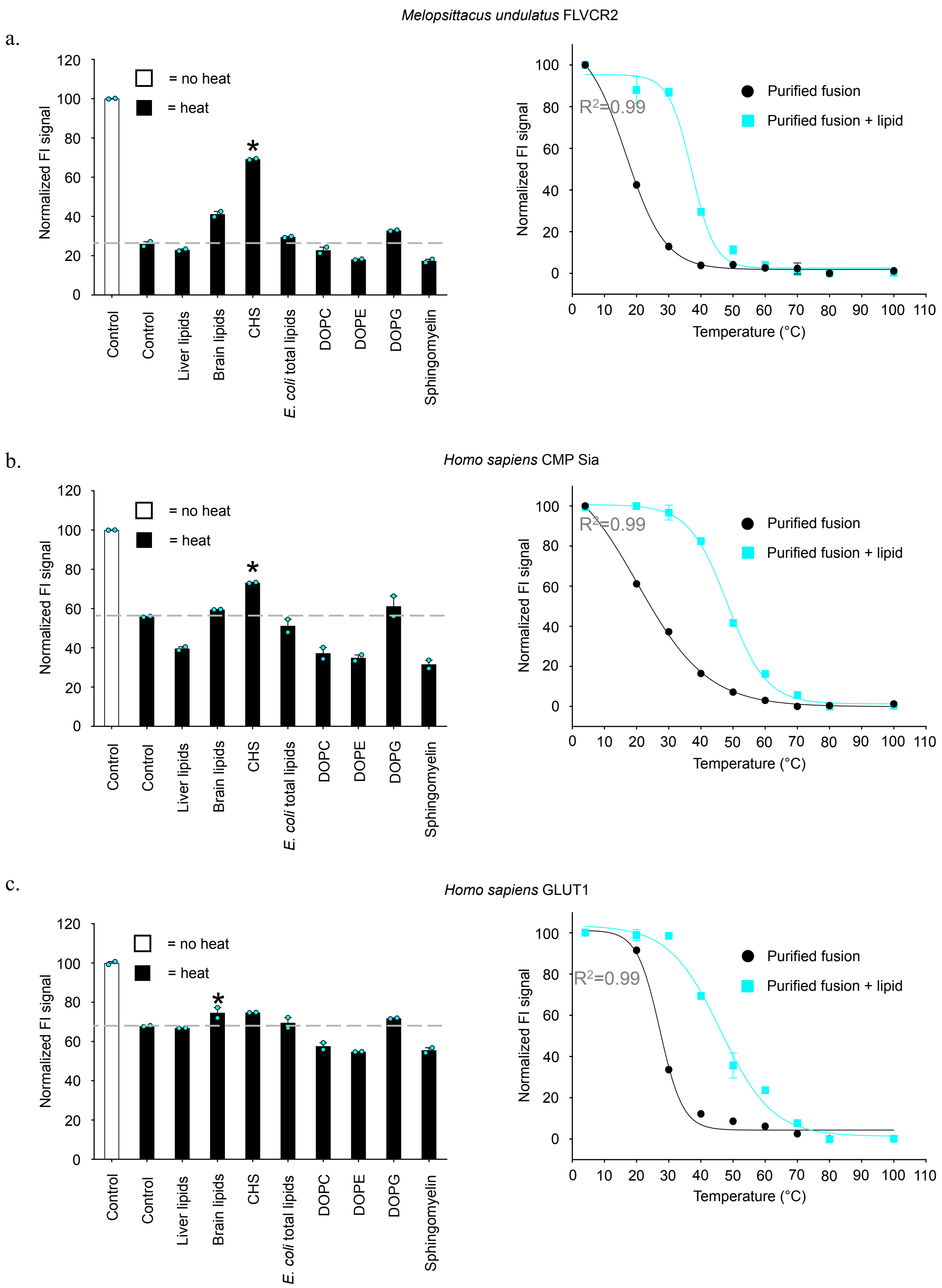

Supplementary Fig. 9 Screening for lipid stabilization of purified eukaryotic membrane proteins using GFP-TS. Left: Supernatant fluorescence of purified fusion before heating T_m + 5°C (non-filled bars) and that remaining after heating and centrifugation (black bars) in the presence of listed lipids: **a.**, Bird FLVCR2 **b.**, Human CMP-Sia **c.**, Human GLUT1; the asterisk indicates the most stabilizing lipid (bars show the range of two technical replicates.

Right: GFP-TS melting curves for purified fusions in the absence (black) and presence of the most stabilizing lipid (cyan) as identified by screen; error bars show the range of two technical replicates and the values reported for the apparent T_m are the mean ± s.e.m. of the fit and are tabulated in **Supplementary Table 1.**

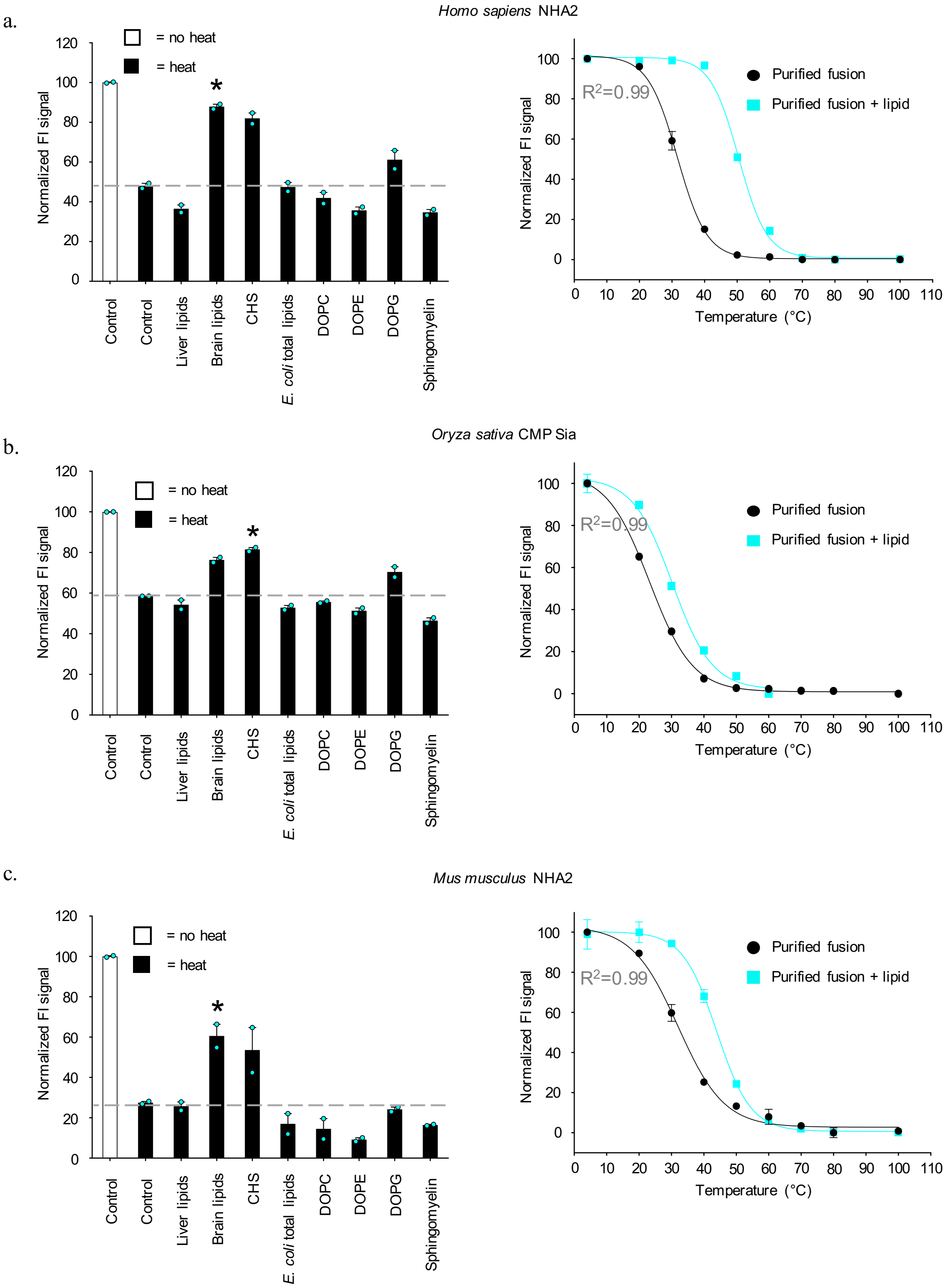

Supplementary Fig. 10 Screening for lipid stabilization of purified eukaryotic membrane proteins using GFP-TS. Left: Supernatant fluorescence of purified fusion before heating T_m + 5°C (non-filled bars) and that remaining after heating and centrifugation (black

bars) in the presence of listed lipids: **a**., Human NHA2 **b**., Plant CMP-Sia and **c**., Mouse NHA2; the asterisk indicates the most stabilizing lipid (bars show the range of two technical replicates. Right: GFP-TS melting curves for purified fusions in the absence (black) and presence of the most stabilizing lipid (cyan) as identified by screen; error bars show the range of two technical replicates and the values reported for the apparent T_m are the mean ± s.e.m. of the fit and are tabulated in **Supplementary Table 1.**

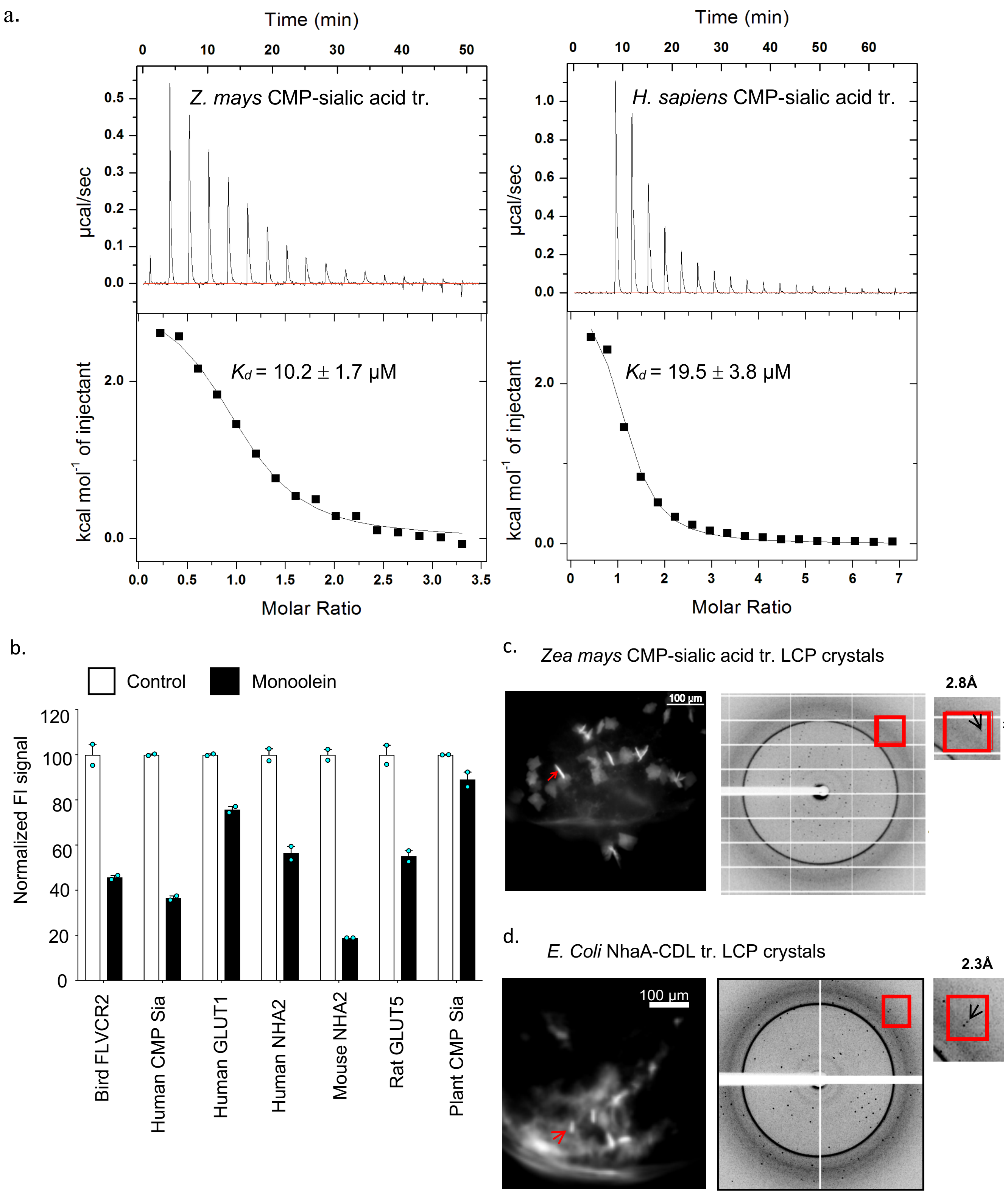

Supplementary Fig. 11. Using GFP-TS for functional and structural characterization of membrane proteins. **a**. Representative ITC thermogram obtained by successive addition of CMP to purified putative plant CMP-sialic acid transporter (left) and human CMP-sialic acid

transporter (right) (see Methods). Integrated heats with baseline-subtracted (red line) inverted power data are shown in top panels. Fit to a single binding site mechanism with a fixed 1:1 stoichiometry is shown in bottom panels (black line). K_d values are reported as the mean ± s.e.m. of the fit. **b**. Supernatant fluorescence of purified fusion before heating T_m + 5°C (non-filled bars) and that remaining after heating and centrifugation (black bars) in the presence of monoloein (error bars show the range of two technical replicates). **c**. Lipidic cubic phase crystals of NhaA shown under a UV-microscope that were obtained in monoolein when 2 % (w/v) cardiolipin 18:1 was added during the final stages of purification prior to crystallization (left) and X-ray diffraction image of an LCP crystal of NhaA (right). **d**. as in c. for LCP crystals of *Zea mays* CMP-sialic acid transporter when CMP was added prior to crystallization (left) and X-ray diffraction image (right).
